# Impact of 6-Month Exposure to Aerosols From Potential Modified Risk Tobacco Products Relative to Cigarette Smoke on the Rodent Gastrointestinal Tract

**DOI:** 10.3389/fmicb.2021.587745

**Published:** 2021-07-02

**Authors:** James N. D. Battey, Justyna Szostak, Blaine Phillips, Charles Teng, Ching Keong Tung, Wei Ting Lim, Ying Shan Yeo, Sonia Ouadi, Karine Baumer, Jerome Thomas, Jacopo Martinis, Nicolas Sierro, Nikolai V. Ivanov, Patrick Vanscheeuwijck, Manuel C. Peitsch, Julia Hoeng

**Affiliations:** ^1^PMI R&D, Philip Morris Products S.A., Neuchâtel, Switzerland; ^2^PMI R&D, Philip Morris International Research Laboratories Pte. Ltd., Science Park II, Singapore, Singapore

**Keywords:** microbiome, smoking, THS 2.2, CHTP 1.2, heat-not-burn, next-generation sequencing, ApoE

## Abstract

Cigarette smoking causes adverse health effects that might occur shortly after smoking initiation and lead to the development of inflammation and cardiorespiratory disease. Emerging studies have demonstrated the role of the intestinal microbiome in disease pathogenesis. The intestinal microbiome is susceptible to the influence of environmental factors such as smoking, and recent studies have indicated microbiome changes in smokers. Candidate modified risk tobacco products (CMRTP) are being developed to provide substitute products to lower smoking-related health risks in smokers who are unable or unwilling to quit. In this study, the ApoE^–/–^ mouse model was used to investigate the impact of cigarette smoke (CS) from the reference cigarette 3R4F and aerosols from two CMRTPs based on the heat-not-burn principle [carbon-heated tobacco product 1.2 (CHTP 1.2) and tobacco heating system 2.2 (THS 2.2)] on the intestinal microbiome over a 6-month period. The effect of cessation or switching to CHTP 1.2 after 3 months of CS exposure was also assessed. Next-generation sequencing was used to evaluate the impact of CMRTP aerosols in comparison to CS on microbiome composition and gene expression in the digestive tract of mice. Our analyses highlighted significant gene dysregulation in response to 3R4F exposure at 4 and 6 months. The findings showed an increase in the abundance of *Akkermansiaceae* upon CS exposure, which was reversed upon cessation. Cessation resulted in a significant decrease in *Akkemansiaceae* abundance, whereas switching to CHTP 1.2 resulted in an increase in *Lactobacillaceae* abundance. These microbial changes could be important for understanding the effect of CS on gut function and its relevance to disease pathogenesis *via* the microbiome.

## Introduction

The composition and stability of the gastrointestinal microbiome is known to be closely linked to the health of its host (Sommer and Bäckhed, 2013). Perturbation of the gut microbiome to a state termed dysbiosis is often associated with disease. This can occur because of chemical insult—which has led to increased interest from the toxicology community—and it has been suggested that understanding the impact of potential toxicants on the gut microbiome is necessary to gain a full understanding of their physiological effects on the host (Licht and Bahl, 2018). The intestinal microbiome, in particular, not only plays an essential role in digestion of food and synthesis of key metabolites and vitamins, but is also implicated in a number of different disease states, including diabetes and neurological disorders. Recent studies have demonstrated the role of the intestinal microbiome in disease pathogenesis (Magami et al., 1990; Bibiloni et al., 2006; Bringiotti et al., 2014; Scotti et al., 2017). In particular, it has been linked to inflammation, immune status, and gut boundary integrity; changes in intestinal microbiota has also been observed in subjects with obesity (Turnbaugh et al., 2009; Henao-Mejia et al., 2012), inflammatory bowel disease (Palm et al., 2014), colorectal cancer (Gagniere et al., 2016), and diabetes (Qin et al., 2012). The composition of the microbiome has recently also been linked to atherosclerotic cardiovascular disease status (Jie et al., 2017).

The intestinal microbiome is susceptible to the influence of environmental factors such as smoking, and recent studies have indicated microbiome changes in smokers (Charlson et al., 2010; Brook, 2011; Savin et al., 2018). Because not all smokers necessarily stop smoking cigarettes, alternative modified risk tobacco products (MRTP) are being developed to provide substitute products for smokers who are unable or unwilling to quit smoking. The United States Food and Drug Administration defines an MRTP as “any tobacco product that is sold or distributed for use to reduce harm or the risk of tobacco-related disease associated with commercially marketed tobacco products” (Food and Drug Administration, 2012; FDA, 2015). Carbon Heated Tobacco Product (CHTP) 1.2 and Tobacco Heating System (THS) 2.2 are two such heat-not-burn tobacco products developed by Philip Morris International (PMI) (Smith et al., 2016; Phillips et al., 2018).

Here, we present an analysis of the gastrointestinal aspect of a previously reported study which used the ApoE^–/–^ mouse model to assess the respiratory and cardiovascular impact of these products relative to cigarette smoke (CS) exposure (Phillips et al., 2019). In this study, ApoE^–/–^ mice were exposed to CS from a 3R4F reference cigarette or aerosols from two candidate modified risk tobacco products (CMRTPs), THS 2.2 or CHTP 1.2, over a 6-month period. Here, we examine the effect of these products on the gastrointestinal tract, with particular focus on the microbiome. We investigated the long-term effects of exposure, as well as the effects specifically of smoking cessation and switching to CHTP 1.2 aerosol after 3 months of CS exposure.

## Materials and Methods

### General Study Design

The general study design has been described previously (Phillips et al., 2019) and is recapitulated here for completeness. Briefly, female ApoE^–/–^ mice were randomized into the groups shown in Supplementary Figure 1. The sham group was exposed to filtered air, the 3R4F group to CS from the 3R4F reference cigarette (600 μg total particulate matter [TPM]/L aerosol; target exposure concentration equivalent to 28 μg nicotine/L), the CHTP 1.2 group to aerosol from CHTP 1.2 (nicotine levels matched to those of 3R4F CS equivalent to 28 μg nicotine/L), and the THS 2.2 group to aerosol from THS 2.2 (nicotine levels matched to those of 3R4F CS equivalent to 28 μg nicotine/L). The cessation and switch CHTP 1.2 groups were initially exposed to 3R4F CS (600 μg TPM/L aerosol) for 3 months and then to filtered air (cessation group) or CHTP 1.2 aerosol (switch CHTP 1.2 group; nicotine levels matched to those of 3R4F CS equivalent to 28 μg nicotine/L). Dissection time points were after months 3, 4, and 6 (see Supplementary Figure 1).

#### Reference Cigarettes, CMRTPs, and Test-Atmosphere Generation

Mainstream CS was generated from 3R4F cigarettes (University of Kentucky^1^) on a 30-port rotary smoking machine as described previously (Phillips et al., 2015). Aerosols from CHTP 1.2 and THS 2.2 sticks were generated on modified 30-port rotary smoking machines equipped with the respective stick holders (Phillips et al., 2016, 2018). The atmosphere in the aerosol exposure chambers was monitored as described previously; for a detailed description of the procedures, see Phillips et al. (2016) and Phillips et al. (2015). Datasets corresponding to this study can be accessed at: https://doi.org/10.26126/intervals.8lafdu.1. For additional details on animal housing, randomization, and acclimatization, see publications on previous studies (Boue et al., 2012, 2013; Phillips et al., 2015); group sizes were based on a previously proven statistical design (Boue et al., 2012, 2013; Phillips et al., 2015, 2016) in order to minimize the number of animals while maintaining statistical power.

#### Animal Care and Welfare

All procedures involving animals were performed in a facility accredited by the Association for Assessment and Accreditation of Laboratory Animal Care International and licensed by the Agri-Food and Veterinary Authority of Singapore, with approval from an Institutional Animal Care and Use Committee (IACUC protocol #15038) and in compliance with the National Advisory Committee for Laboratory Animal Research Guidelines on the Care and Use of Animals for Scientific Purposes (NACLAR, 2004). Female B6.129P2-Apoe^tm1Unc^ N11 ApoE^–/–^ mice bred under specific pathogen-free conditions were obtained from Taconic Biosciences (Germantown, NY, United States). The age and health status of the mice on arrival was verified by using the health check certificate provided by the breeder. Additional health checks were conducted on live animals (six animals prior to the study start and again at study completion; health screening panel 450 M) and by using serum samples (month 3; health screening panel SM246; Envigo, Hillcrest, United Kingdom).

#### Cage-Based Fecal Sampling, In-Life Study Phase

At months 1, 2, 3, 4, and 5, samples were collected from animals in the month 6 dissection group. Initially there were at least eight cages per exposure group (6–8 animals per cage); in the continuous exposure groups (CHTP, THS, and Sham), six cages were used at time points 4 and 5 (see Supplementary Table 1 for details). Fecal pellets were collected the day after a scheduled cage change (so that no sample would remain in the cage for more than 24 h). The samples were collected at 4-week (28-day) intervals (±2 days) starting from day 33. From each cage, nine pellets were collected and distributed into three microfuge tubes. The tubes were then placed in a freezer box atop of a bed of dry ice and transferred to an ultralow freezer (<−70°C) for storage until shipment to the test site (or biostorage) for processing. For cage-based sampling, all month six groups were selected in order to ensure that the same cages were sampled throughout the study. Mice dedicated for individual molecular analysis were selected and processed as described previously (Phillips et al., 2016). Samples were then stored at −80°C until further processing (Supplementary Figure 2).

#### Samples Collected During Necropsy

At necropsy (Supplementary Table 2), the cecum was isolated and separated from the intestine and colon. Cecal contents were then removed and transferred to a 2-mL safe-lock tube. The remaining cecal tissue was then opened longitudinally with a cut on one side and rinsed in phosphate-buffered saline to remove any remaining cecal contents. The tissue was then snap-frozen in a MagnaLyser tube and stored at <−70°C until further processing. When present, fecal pellets were also removed at necropsy. Up to two pellets were removed from the colon and transferred to a 2-mL RNAse-free tube, snap-frozen, and stored at <−70°C (before being shipped to biostorage/Neuchâtel for storage or analysis).

### DNA Isolation and Sequencing

#### Zymobiomics Library Preparation

DNA was extracted from fecal samples by using a ZymoBIOMICS DNA Miniprep kit (cat. no. D4300; Zymo Research, Irvine, CA, United States) and protocol version 1.2.2. The samples were ground by using a MagNA Tissue Lyser (Roche, Basel, and Roche), and the extracted DNA was quantified on a Qubit 2.0 fluorometer (Thermo Fisher Scientific, Waltham, MA, United States). The DNA was sheared by using a Covaris E220 focused ultrasonicator (Matthews, NC, United States), and DNA sequencing libraries were prepared by using the NuGEN Ovation Ultralow system V2 kit (San Carlos, CA, United States).

#### Qiagen Library Preparation

DNA was extracted from fecal samples by using the QIAamp Fast DNA Stool Mini Kit (QIAGEN, Hilden, Germany). The QIAamp Stool Pathogen Detection protocol was used with a modified lysis protocol in which samples were ground by using a steel bead (Qiagen), homogenized by vortexing, lysed for 5 min at 70°C, vortexed again, and incubated for 2 min at 95°C. The extracted DNA was quantified on NanoDrop1000 (Thermo Fisher Scientific, Waltham, MA, United States). The DNA was sheared by using a Covaris E220 focused ultrasonicator, and DNA sequencing libraries were prepared by using the NuGEN Ovation Ultralow system V2 kit.

#### Cecum RNA Seq Library Preparation

The tissue was ground by using the MagNA Tissue Lyser. RNA extraction was performed by using the miRNeasy Mini Kit on a QIAcube (Qiagen, Hilden, Germany). Quantification was done on a NanoDrop1000 and quality control on a Bioanalyzer by using the Agilent RNA 6000 Nano Kit. DNA sequencing libraries were prepared by using the TruSeq Stranded mRNA sample prep kit (Illumina, San Diego, CA, United States).

#### Sequencing

Normalized libraries were pooled into multiplexes and clustered on Illumina HiSeq 3000/4000 PE flow cells by using Illumina HiSeq 3000/4000 PE Cluster Kits (Illumina). Sequencing was performed on an Illumina HiSeq 4000 system by using Illumina HiSeq 3000/4000 SBS kits (300 cycles).

### Bioinformatics

#### RNA Sequencing Data Processing

For RNA Seq read analysis, raw reads were mapped directly to the mouse genome assembly (GRCm38) without filtering, by using Hisat2 (Sirén et al., 2014) version 2.1.0. Reads were counted by using the “count” program from the HTSeq suite (Anders et al., 2015) version 0.11.0. Differential gene expression was determined by using Deseq2 (Love et al., 2014) version 1.18.1, whereby normalization and calculation of differential expression were performed at each time point separately. The differential gene expression for each exposure condition was calculated relative to the sham exposure group at each time point.

#### DNA Sequencing Data Processing

Sequencing reads were cleaned of adapters (minimum length after trimming, 100 bases) and trimmed to a maximum length of 150 bases. After cleaning, reads were mapped in sequence to the mouse genome assembly (GRCm38), the human genome (GRCh38), a viral sequence collection, and a collection of plasmid sequences. At each step, only read pairs were retained for which neither read could be mapped. After cleaning, trimming, and screening, the reads were mapped to a reference database of “chromosome” and “complete genome” sequences from bacteria, archaea, and protists, which was obtained by querying the NCBI Assembly website^2^. Read mapping was performed by using minimap2 (Li, 2018) version 2.8. Reads which were flagged as “properly paired” and had a mapping quality value of over five were retained and counted. Samples counts were merged, and taxonomic information from the NCBI taxonomy was added in post-processing. Samples with fewer than 100,000 mapped sequence reads were removed from the analysis (one sample in case of the cage-wise ZymoBIOMICS data in the CHTP group, 2 months). Ultimately, only bacterial taxa were chosen for downstream analysis. In order to avoid probable mapping artifacts, low-abundance taxa were removed: The counts table was filtered by retaining only the most abundant species, which together accounted for 95% of the total average mapped read number. After this step, analysis at the higher ranked taxon levels was performed.

#### Statistical Analysis

Individual-level analyses and cage-level analyses were performed in R (R Core Team, 2018) version 3.4.3 and 3.5.1 respectively, using DESeq2 (Love et al., 2014) version 1.18.1 and 1.22.2 respectively. For the microbiome analysis, differential taxon abundance was estimated analogously to the gene expression data, but using microbial taxon counts instead of gene counts. For the stool samples collected by cage, differential abundance was modeled based on a simple additive model of exposure and time point (both expressed in R as factors):

Abundance∼Exposure+Timepoint

For the long term effects model (months 1–5) the exposure contrasts are relative to the sham group and the time contrasts are relative to month 1. For the switching effect model (months 3–5), the exposure contrast is relative to the CS exposure group and the time contrasts are relative to month 3. The full results from the Deseq2 analysis are presented in the Supplementary Data Sheet 2.

Phylogenetic trees were produced by using the GraPhlAn software (Asnicar et al., 2015).

## Results

### Cage-Wise Effects

**FIGURE 1:**
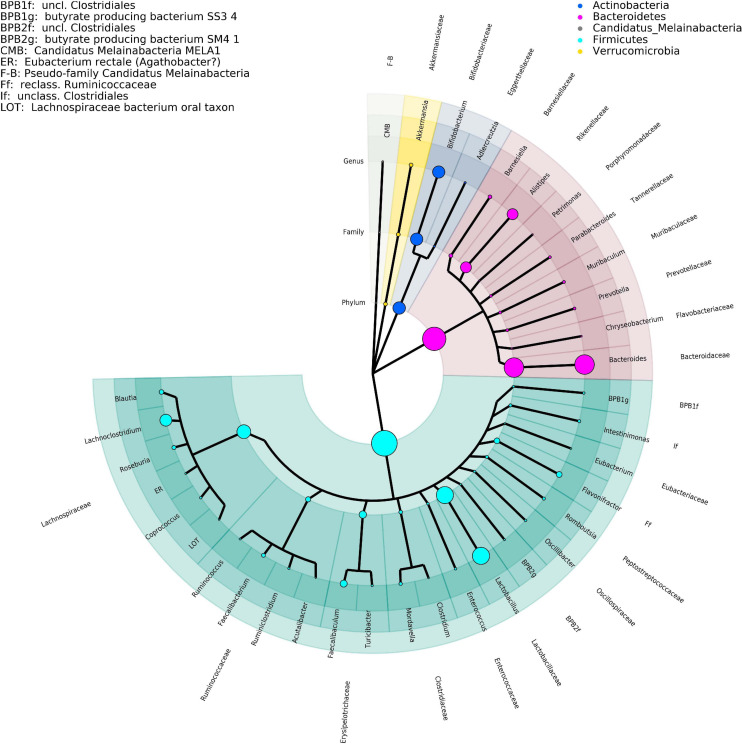
Phylogenetic tree representing the major taxa appearing in the time-series analysis performed by DNA isolation method 1. Phylogeny data are taken from the NCBI taxonomy database, where only the taxonomic ranks of phylum, family, and genus are shown. The size of the clade markers is indicative of the average normalized read abundance for the taxon. Pseudofamilies and pseudogenera are used for organisms defined at a lower taxonomic level but lacking higher ranking categorization.

The effect measured here are the changes over time measured per cage. For a brief overview of the key taxa, please consult the figures section (Figure 1).

#### Long-Term Effects of CS and CMRTP Aerosol Exposure

**FIGURE 2:**
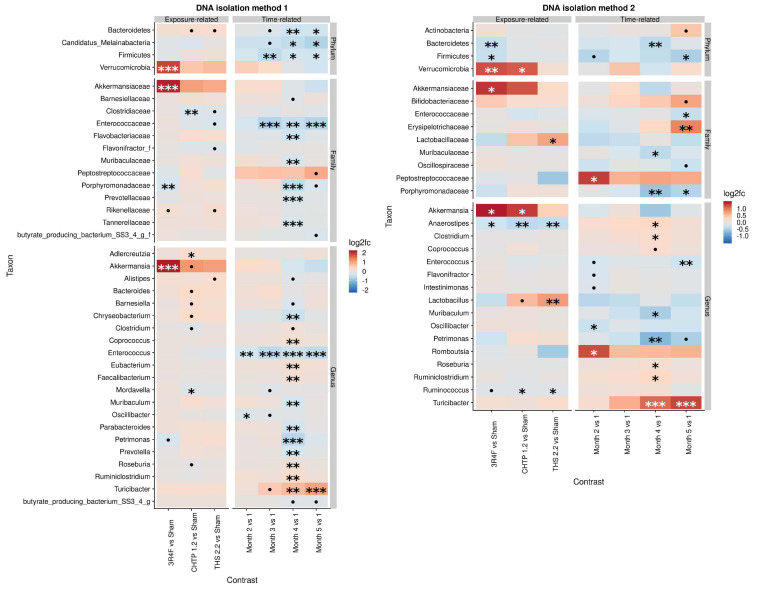
Effects of 5-month continuous exposure on the fecal microbiome composition (excluding the switching groups) based on cage-wise sampling. Fold changes in the abundance of various microbial taxa (y-axis) are modeled as a function of exposure type relative to the sham group and time point relative to the first time point (month 1). Two methods of DNA isolation were used for different samples collected from the cages. Significance values are indicated as follows: ***p ≤ 0.001; **0.001 < *p* ≤ 0.01; *0.01 < *p* < 0.05; *0.05 < *p* < 0.1.

The effect of exposure type on microbiome composition was determined by modeling differential abundance as a function of aerosol exposure type relative to sham exposure as well as a function of time relative to the reference time point, which in this case is month 1. All treatments groups showed significant modifications in the fecal microbiome over 5 months (Figure 2). Analysis with DNA isolation method 1 (ZymoBiomics) revealed relatively few but highly significant differences between sham and CS exposure. At the phylum level, a significant increase in Verrucomicrobia abundance was observed, which could be traced to *Akkermansia* in the lower taxonomic rank of genus, and at the family level, a decrease in *Porphyromonadaceae* abundance was observed. In the CHTP 1.2 group, *Clostridiaceae* were decreased in abundance at the family level; at the genus level, the abundance of *Adlercreutzia* was increased, while that of *Mordavella* was decreased. The THS 2.2 exposure group showed no significant changes, although some statistically insignificant changes were observed, for example, in *Clostridiaceae* abundance. In terms of time dependence, two changes at the genus level were significant at more than one time point, notably in the decreased abundance of *Enterococcus* over time and increased abundance of *Turicibacter*.

The findings from DNA isolation method 2 (Qiagen) showed a similar trend for Verrucomicrobia as observed for method 1: they were more abundant relative to the sham in the CS group as well as in the CHTP 1.2 group. Another significant difference in abundance at the family level was in *Lactobacillaceae* abundance, which was significantly increased in the THS 2.2 group, but reduced, albeit not significantly, in the CS group; this trend was also seen at the corresponding genus level (*Lactobacillus*). Otherwise, all three exposure groups experienced a decrease in the abundance of genera *Aaerostipes* and *Ruminococcus*, although the latter was not statistically significant in the CS group. The findings of method 1 showed the time dependence of *Turicibacter* abundance: It gradually increased over time and became significant at months 4 and 5. The *Peptostreptococcaceae* were generally increased in abundance over the time course, though significantly only at month 2.

#### Similarity of the Three CS Exposure Groups in the Short Term

To analyze the cessation effect, the two intervention groups (switch CHTP 1.2 and cessation) were modeled in the same way as before, only by using 3R4F as the reference exposure and month 1 as the reference for initial exposure and month 3 (i.e., the intervention time point) for switching/cessation analysis. From months 1 through 3, there were no significant differences in bacterial taxon abundances (Supplementary Figure 3) between those groups exposed to 3R4F aerosol (i.e., the CS, cessation and switching groups). For DNA isolation method 1, some time-dependent effects were similar to those before, but somewhat more pronounced: The significance of change in Verrucomicrobia abundance was more noticeable, as was the decrease in Firmicutes abundance. With DNA isolation method 2, there were some similarities in the observed changes in microbial abundances, but furthermore some changes were apparent, which were not observed with method 1: The abundance of Verrucomicrobia was observed to increase over time with both methods, but Actinobacteria abundance change was only observed for method 2; upon tracing this change to lower taxonomic ranks, it appears that *Bifidobacteria* are responsible for this trend.

#### Cessation/Switching

**FIGURE 3:**
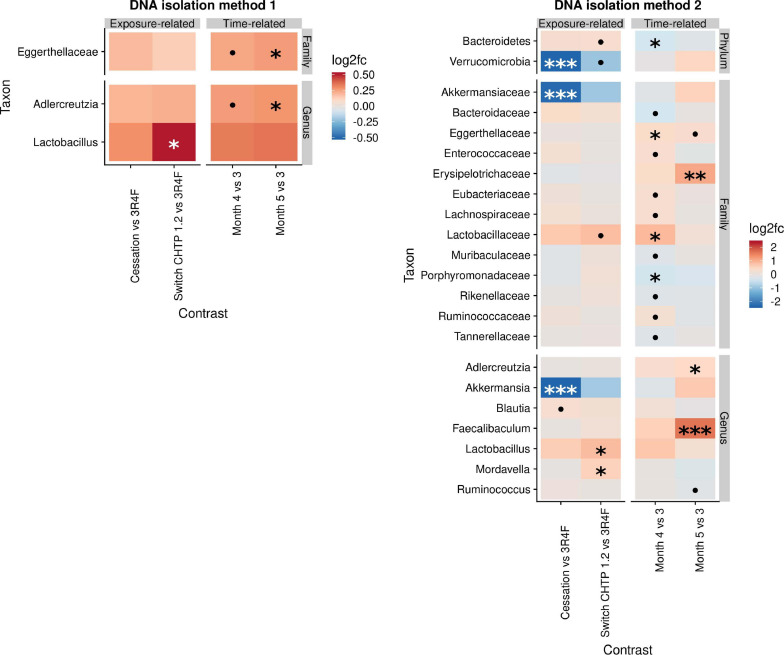
Effects of cessation/switching to CHTP 1.2 relative to continued smoke exposure (lower). The intervention time point is at month 3. Fold-changes in the abundance of the various microbial taxa (y-axis) were modeled as a function of the exposure type relative to the CS exposure group, and the time point relative to the cessation/switching time point at month 3. Two methods of DNA isolation were used on different samples taken from the cages. Significance values are indicated as follows: ***p ≤ 0.001; **0.001 < *p* ≤ 0.01, *0.01 < *p* < 0.05; *0.05 < *p* < 0.1.

The effects of cessation and switching to CHTP 1.2 were modeled by contrasting the fold changes in these two groups with those in the continued CS exposure group and by contrasting the time-course development relative to the switching time point at month 3 (Figure 3). This part of the analysis, therefore, shares time point 3 with the above analysis. The findings of DNA isolation method 1 revealed very few changes upon cessation and switching, with the main one being an increase in *Lactobacillus* abundance in the switch CHTP 1.2 group. According to the results of DNA isolation method 2, *Lactobacillus* abundance was also increased in both the switch and cessation groups, albeit only to a non-significant degree in the latter. While *Akkermansia* abundance in the cessation group was strongly and significantly decreased, it was also decreased in the switch group, albeit not significantly.

### Individual-Level Effects

The differential abundance of the various taxa in the fecal microbiome was analyzed for each exposure group and at each time point (Supplementary Figure 4). In fecal matter, differential abundance was apparent in many cases; however, very few of these changes were statistically significant according to the model. The variability of individual sample measurements simply outweighed the signal that could be seen in the cage-wise analysis. In cecal digesta (Supplementary Figure 4), the effect of exposure was slightly more pronounced, implying either that the cecum is more sensitive to the effect of CS than the colon or that the exposure effect diminishes along the length of the digestive tract. To investigate the similarity of the findings from cecal and fecal matter, the normalized abundances of the most important taxa from both sample types were modeled as a linear model of each other (Supplementary Table 3). Key families with extremely similar abundances in both sample types included *Akkermansiaceae*, *Erysipelotrichaceae*, and *Bifidobacteriaceae*.

### Cecum Gene Expression Analysis

**FIGURE 4:**
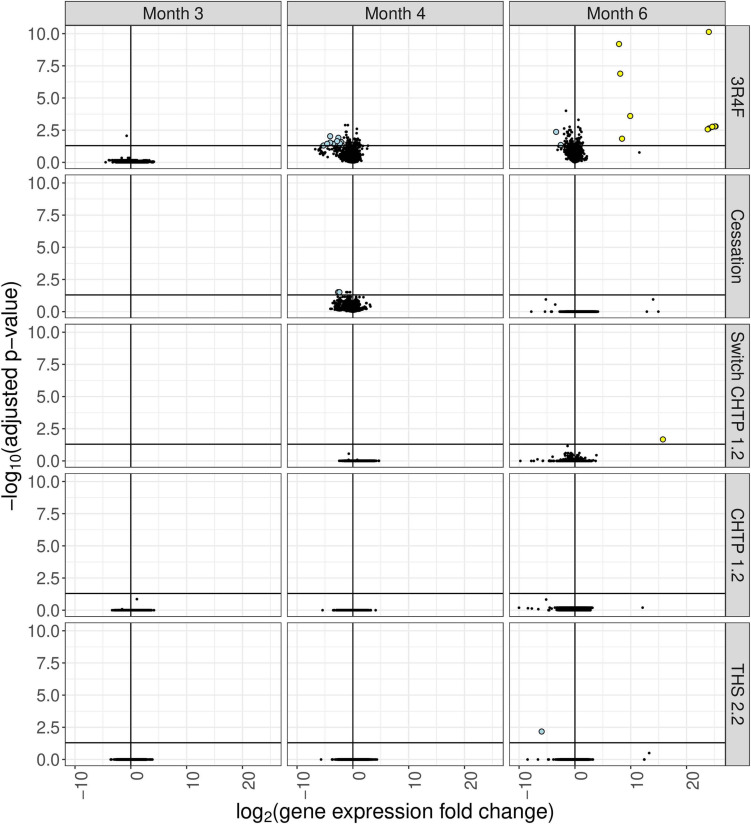
Differential gene expression analysis of cecum tissue from mice in the various exposure groups, displayed as a “volcano plot.” Each data point corresponds to a gene, with the x-axis value being the log2 fold change in the exposure group relative to the sham group, and the y-axis being the negative log10 of the adjusted *p* value associated with the fold change. Points with adjusted *p* values <= 0.05 and an absolute expression fold change greater than 2 are rendered as large circles and are colored by whether they have an expression fold change less than –2 (light blue) or greater than 2 (yellow).

**FIGURE 5:**
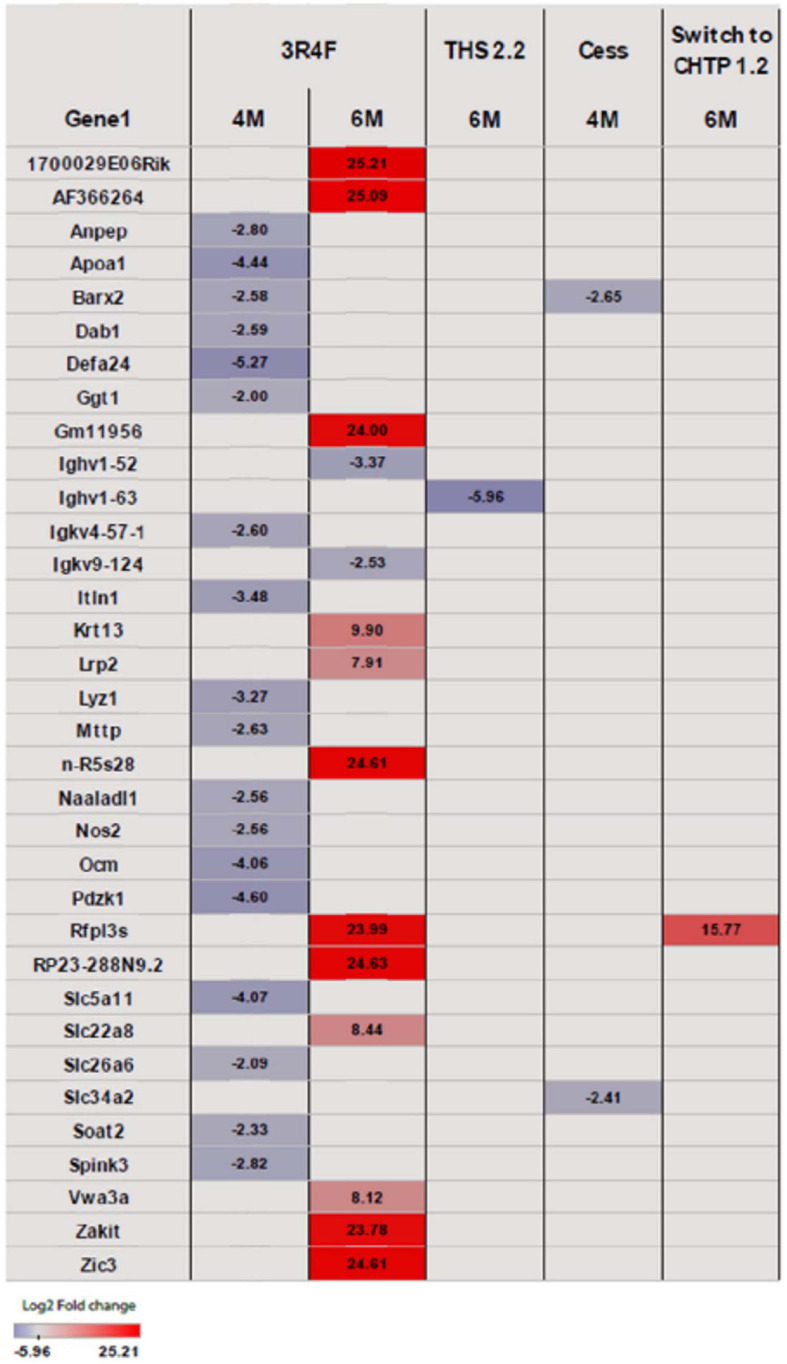
Heat map representing differentially expressed genes in the cecum. Blue indicates significantly (*p* <= 0.05) decreased expression, and red indicates significantly increased expression. The intensity of coloring varies with log2 fold change.

Exposure to 3R4F CS impacted the cecum transcriptome mostly at the later time points (4 and 6 months; for a general overview, see Figure 4; for specific genes, see Figure 5). After 4 months of exposure to 3R4F CS, 18 genes were significantly downregulated in the cecum. *Defa24* (defensin, alpha, 24), *Pdzk1* (PDZ domain containing 1), *Apoa1* (apolipoprotein A1), *Ocm* (oncomodulin), and *Slc5a11* (solute carrier family 5 member 11) were the top five downregulated genes at month 4 of 3R4F CS exposure. At month 6 of exposure to 3R4F CS, 14 genes were significantly dysregulated (12 were upregulated, and two were downregulated). While *1700029E06Rik* (RIKEN cDNA gene), *Af366264* (cDNA sequence), and *Zic3* (zinc finger protein of the cerebellum 3) were significantly upregulated, *Ighv1-52* (immunoglobulin heavy variable 1–52) and *Igkv9-124* (immunoglobulin kappa chain variable 9–124) were significantly downregulated. In mice exposed to CMRTP aerosols, the cecum tissue analysis showed one differentially expressed gene at the 6-month time point in the THS 2.2 group (relative to the sham group) and no differentially expressed genes in the CHTP 1.2 group. Smoking cessation restored the transcriptome to a level close to that of the sham group after 3 months. Only two genes—*Barx* (BarH-like homeobox 1) and *Slc34a2* (solute carrier family 34 member 2)—appeared significantly dysregulated in the cessation group at 4 months of exposure (i.e., after 1 month of smoking cessation). Similar to cessation, switching to CHTP 1.2 restored the transcriptome to a state similar to that of the sham group. At the 6-month time point, only one gene, *Rfpl3s* (RIKEN cDNA 4930563M21 gene), remained dysregulated in the cecum.

## Discussion

In this study, we compared, at nicotine-matched concentrations, the impact of CMRTP aerosols with that of 3R4F CS on the microbiome and cecum gene expression in ApoE^–/–^ mice.

### Changes Observed in the Cage-Wise Microbiome Analysis, and Their Consistency Between Methods

For the long-term analysis, we examined the absolute effects of chronic exposure to aerosols over 5 months relative to sham exposure. We did this by using cage-wise samples, which were analyzed by two different DNA isolation methods. The most predominant effect of 3R4F exposure was the significant increase in the abundance of the Verrucomicrobia phylum, which was demonstrated by both DNA isolation methods. A notable difference between the exposure types was the increase in *Lactobacillus* abundance, according to method 2, in the non-CS exposure groups; the effect was significant in the THS 2.2 group and near significant in the CHTP 1.2 group, while there was a non-significant decrease in the 3R4F group. The consequence of the time-dependent increase in Turicibacter is unclear, as Turicibacter is not a highly studied genus, however, recent research has shown a link between at least one species of the genus and serotonin metabolism in the gut of mice (Fung et al., 2019). The effect does not seem to be dependent on any particular exposure regimen, and is probably an age-related effect.

In the short term, all three groups were initially exposed to CS, and the two non-CS exposure groups were analyzed relative to the CS exposure group. There was no significant difference among the groups up to the switching/cessation time point at month 3, indicating good reproducibility among replicate groups. There were some short-term time-dependent changes, such as a decrease in *Enterococcus* abundance; this effect was somewhat reproducible in both DNA isolation methods in the sense that the trend was in the same direction of change, although the effect was not deemed significant for both methods simultaneously. For the switching/cessation phase, the switching group showed two conspicuous changes. The first was a decrease in Verrucomicrobia abundance in the non-CS exposure groups (relative to the CS exposure group), which was highly significant in the cessation group and close to significant in the CHTP 1.2 group. The other was the increase in *Lactobacillus* abundance in both non-CS exposure groups, although the increase was significant only in the CHTP 1.2 group.

The findings of many studies are often influenced by the method used (Poussin et al., 2018), and, therefore, congruence among different methods is essential. In the present study, the major effects of the exposures were observed consistently between the two DNA isolation methods, however, there were numerous effects observed only for one of the two methods. There are two reasons for which there might be inconsistencies between the methods. For one, cage-wise sampling involves collecting samples that might not always be from the same animal and might even be a mixture of samples from multiple animals. This means that inter-animal variation cannot be accounted for in cage-wise sampling, and, thus, random variability among the samples is higher than necessary. The other source of discrepancy is the materials and reagents of the kits, which might have different efficacies in isolating DNA from various microbial taxa. Despite these differences, in the present study, the key changes were reproducible in both methods, namely the increase in Verrucomicrobia abundance in the CS group and increase in *Lactobacillus* abundance in the CHTP 1.2 switch group. In our analysis, the Qiagen and ZymoBiomics kits both detect the changes in Verrucomicrobia abundance and also show agreement on other changes such as the presence of *Lactobacillaceae* in the switching/cessation phase.

With regard to the biological implications of these findings, recent studies have demonstrated that smoking not only affects the tissues and organs of the human body but also influences and alters the gut microbiota (Biedermann et al., 2014; Allais et al., 2016). *Akkermansiaceae* is a family of mucin-degrading bacteria that are commonly found in the mammalian gut; these bacteria are usually more abundant in the gut of healthy subjects than in that of diabetic and obese patients (Santacruz et al., 2010; Karlsson et al., 2012; Tilg and Moschen, 2014) or patients with bowel diseases (Png et al., 2010). This stands in contrast to our previous analysis, wherein we demonstrated that 3R4F CS exposure causes lung inflammation, emphysematous changes, and an increase in atherosclerotic plaque area, consistent with the notion that exposure to 3R4F CS is associated with a diseased status. In response to CMRTP aerosol exposure, *Akkermansiaceae* abundance was not as strongly increased after 3 or 5 months post-exposure, which suggests that the impact of CMRTP aerosols on the gut microbiome is less pronounced than that of 3R4F CS. A similar effect of smoke exposure on *Akkermansia* abundance has been shown in other studies (Tam et al., 2020), who observed this effect most strongly in male mice. By contrast, the present study produces the effect using only female mice. At the host transcriptome level, our investigation demonstrated a prominent dysregulation of cecum genes in response to 3R4F CS, but no significant changes in response to CMRTP aerosols, cessation, or switching. Gene expression analysis showed a noticeable impact on repression after 4 months of exposure and activation at 6 months of exposure, suggesting the activation of a cascade of different mechanisms and pathways during long-term exposure.

### Potential Link to Inflammatory Bowel Diseases

Cigarette smoke has been shown to influence the severity and progression of the two most frequent types of IBD, Crohn’s disease (CD) and ulcerative colitis (UC). Smoking has been associated with poorer prognosis of CD and worse quality of life (Cosnes, 2010). Holdstock et al. (1984) reported that CD patients with active smoking habits have an increased incidence of disease relapse and more severe pain. In contrast, there is an inverse association between active smoking and development of UC (Birrenbach and Bocker, 2004), including effects on disease risk, progression, and relapse rate. *Akkermansiaceae* are known to be mucinophiles, residing in and feeding off mucus, which is a part of the protective gut barrier. Although no direct measurement of mucus layer thickness was made in this study, the increased abundance of *Akkermansiaceae* could indicate that smoke exposure causes either an increase in mucus production or a change in the composition of mucus, at least the latter of which has been observed in chronically smoke-exposed mice (Verschuere et al., 2012). This could be one of the links to explain the observed inverse relationship between cigarette smoking and UC development; smoke-induced changes to the mucus layer have been observed in other animal models (Verschuere et al., 2012) and might be linked to this effect (Savin et al., 2018). Furthermore, the various changes due to smoke exposure suggest that some of these bacteria might be early indicators for dysbiosis in the gut, which may be linked to the exacerbation of CD by CS.

### Summary

Only relatively small changes were observed in the intestinal microbiome of mice upon CS or CMRTP aerosol exposure. However, some of these changes could be of functional importance in understanding the effect of CS on the gut and its role in influencing various subtypes of inflammatory bowel diseases. Most notably, Akkermansiaceae were often more abundant in samples from the CS-exposed groups than in those from sham aerosol-exposed groups; this bacterium is linked to gut barrier function.

## Data Availability Statement

The datasets presented in this study can be found in online repositories. The names of the repository/repositories and accession number(s) can be found below: https://www.ebi.ac.uk/ena, PRJEB39465.

## Ethics Statement

The animal study was reviewed and approved by Philip Morris International (PMI) Research Laboratories Institutional Animal Care and Use Committee (IACUC protocol 15038).

## Author Contributions

JB performed the computational analysis of sequencing data and wrote manuscript. JS contributed to the study design, analysis of transcriptomics data, and co-wrote the manuscript. BP contributed to the study design and oversaw the execution of *in vivo* study phase. CT, WL, and YY executed the in life phase of the study, comprising animal care and husbandry, aerosol treatment administration during the in life study phase, and data collection and analysis. CKT was responsible for set-up of the exposure apparatus and for aerosol generation and delivery to the exposure chambers. PV managed the laboratory producing performing the *in vivo* experiments. SO, KB, JT, and JM performed DNA and RNA extractions, sequencing library preparation, and sequencing. NS oversaw the execution of the sequencing and analysis. NI managed the laboratory producing the sequencing data. MP and JH conceived the study, contributed to the study design, and prepared the manuscript. All authors contributed to the article and approved the submitted version.

## Conflict of Interest

JB, JS, SO, KB, JT, JM, NS, NI, PV, MP, and JH were employed by the company Philip Morris Products S.A. BP, CT, CKT, WL, and YY were employed by the company Philip Morris International Research Laboratories Pte. Ltd.

## Footnotes

1 http://www.ca.uky.edu/refcig

2 https://www.ncbi.nlm.nih.gov/assembly

## References

[B1] Allais L. Kerckhof F. M. Verschuere S. Bracke K. R. De Smet R. Laukens D. (2016). Chronic cigarette smoke exposure induces microbial and inflammatory shifts and mucin changes in the murine gut. *Environ. Microbiol.* 18 1352–1363. 10.1111/1462-2920.12934 26033517

[B2] Anders S. Pyl P. T. Huber W. (2015). HTSeq—a Python framework to work with high-throughput sequencing data. *Bioinformatics* 31 166–169. 10.1093/bioinformatics/btu638 25260700 PMC4287950

[B3] Asnicar F. Weingart G. Tickle T. L. Huttenhower C. Segata N. (2015). Compact graphical representation of phylogenetic data and metadata with GraPhlAn. *PeerJ* 3:e1029. 10.7717/peerj.1029 26157614 PMC4476132

[B4] Bibiloni R. Mangold M. Madsen K. L. Fedorak R. N. Tannock G. W. (2006). The bacteriology of biopsies differs between newly diagnosed, untreated, Crohn’s disease and ulcerative colitis patients. *J. Med. Microbiol.* 55 1141–1149. 10.1099/jmm.0.46498-0 16849736

[B5] Biedermann L. Brulisauer K. Zeitz J. Frei P. Scharl M. Vavricka S. R. (2014). Smoking cessation alters intestinal microbiota: insights from quantitative investigations on human fecal samples using FISH. *Inflammatory Bowel Dis.* 20 1496–1501. 10.1097/mib.0000000000000129 25072500

[B6] Birrenbach T. Bocker U. (2004). Inflammatory bowel disease and smoking: a review of epidemiology, pathophysiology, and therapeutic implications. *Inflammatory Bowel Dis.* 10 848–859. 10.1097/00054725-200411000-00019 15626903

[B7] Boue S. De Leon H. Schlage W. K. Peck M. J. Weiler H. Berges A. (2013). Cigarette smoke induces molecular responses in respiratory tissues of ApoE^–/–^ mice that are progressively deactivated upon cessation. *Toxicology* 314 112–124. 10.1016/j.tox.2013.09.013 24096154

[B8] Boue S. Tarasov K. Janis M. Lebrun S. Hurme R. Schlage W. (2012). Modulation of atherogenic lipidome by cigarette smoke in apolipoprotein E-deficient mice. *Atherosclerosis* 225 328–334. 10.1016/j.atherosclerosis.2012.09.032 23102783

[B9] Bringiotti R. Ierardi E. Lovero R. Losurdo G. Di Leo A. Principi M. (2014). Intestinal microbiota: the explosive mixture at the origin of inflammatory bowel disease? *World J. Gastrointest Pathophysiol.* 5 550–559. 10.4291/wjgp.v5.i4.550 25400998 PMC4231519

[B10] Brook I. (2011). The impact of smoking on oral and nasopharyngeal bacterial flora. *J. Dent. Res.* 90 704–710. 10.1177/0022034510391794 21558542

[B11] Charlson E. S. Chen J. Custers-Allen R. Bittinger K. Li H. Sinha R. (2010). Disordered microbial communities in the upper respiratory tract of cigarette smokers. *PLoS One* 5:e15216. 10.1371/journal.pone.0015216 21188149 PMC3004851

[B12] Cosnes J. (2010). Smoking, physical activity, nutrition and lifestyle: environmental factors and their impact on IBD. *Dig. Dis.* 28 411–417. 10.1159/000320395 20926865

[B13] FDA (2015). *Comments of the National Institute for Occupational Safety and Health to the Food and Drug Administration (FDA) in Response to Establishment of Public Docket; Electronic Cigarettes and the Public Health Workshop.* Avenue Silver Spring, MA: FDA. Docket no FDA-2014-N-1936.

[B14] Food and Drug Administration (2012). *Modified Risk Tobacco Product Applications: Draft Guidance for Industry.* Avenue Silver Spring, MA: FDA.

[B15] Fung T. C. Vuong H. E. Luna C. D. G. Pronovost G. N. Aleksandrova A. A. Riley N. G. (2019). Intestinal serotonin and fluoxetine exposure modulate bacterial colonization in the gut. *Nat. Microbiol.* 4 2064–2073. 10.1038/s41564-019-0540-4 31477894 PMC6879823

[B16] Gagniere J. Raisch J. Veziant J. Barnich N. Bonnet R. Buc E. (2016). Gut microbiota imbalance and colorectal cancer. *World J. Gastroenterol.* 22 501–518. 10.3748/wjg.v22.i2.501 26811603 PMC4716055

[B17] Henao-Mejia J. Elinav E. Jin C. Hao L. Mehal W. Z. Strowig T. (2012). Inflammasome-mediated dysbiosis regulates progression of NAFLD and obesity. *Nature* 482 179–185. 10.1038/nature10809 22297845 PMC3276682

[B18] Holdstock G. Savage D. Harman M. Wright R. (1984). Should patients with inflammatory bowel disease smoke? *Br. Med. J.* 288:362. 10.1136/bmj.288.6414.362 6419927 PMC1444288

[B19] Jie Z. Xia H. Zhong S.-L. Feng Q. Li S. Liang S. (2017). The gut microbiome in atherosclerotic cardiovascular disease. *Nat. Commun.* 8 845–845. 29018189 10.1038/s41467-017-00900-1 PMC5635030

[B20] Karlsson C. L. Onnerfalt J. Xu J. Molin G. Ahrne S. Thorngren-Jerneck K. (2012). The microbiota of the gut in preschool children with normal and excessive body weight. *Obesity* 20 2257–2261. 10.1038/oby.2012.110 22546742

[B21] Li H. (2018). Minimap2: pairwise alignment for nucleotide sequences. *Bioinformatics* 34 3094–3100. 10.1093/bioinformatics/bty191 29750242 PMC6137996

[B22] Licht T. R. Bahl M. I. (2018). Impact of the gut microbiota on chemical risk assessment. *Curr. Opin. Toxicol.* 15 109–113. 10.1016/j.cotox.2018.09.004

[B23] Love M. I. Huber W. Anders S. (2014). Moderated estimation of fold change and dispersion for RNA-seq data with DESeq2. *Genome Biol.* 15:550. 25516281 10.1186/s13059-014-0550-8 PMC4302049

[B24] Magami Y. Azuma T. Inokuchi H. Kawai K. Hattori T. (1990). Turnover of acinar cells in mouse pancreas–3H-thymidine autoradiographic investigation. *Gastroenterol. Jpn* 25:514. 10.1007/bf02779349 2210231

[B25] NACLAR. (2004). *Guidelines on the Care and Use of Animals for Scientific Purposes National advisory committee for laboratory animal research*, Singapore.

[B26] Palm N. W. de Zoete M. R. Cullen T. W. Barry N. A. Stefanowski J. Hao L. (2014). Immunoglobulin A coating identifies colitogenic bacteria in inflammatory bowel disease. *Cell* 158 1000–1010. 10.1016/j.cell.2014.08.006 25171403 PMC4174347

[B27] Phillips B. Schlage W. K. Titz B. Kogel U. Sciuscio D. Martin F. (2018). A 90-day OECD TG 413 rat inhalation study with systems toxicology endpoints demonstrates reduced exposure effects of the aerosol from the carbon heated tobacco product version 1.2 (CHTP1.2) compared with cigarette smoke. I. Inhalation exposure, clinical pathology and histopathology. *Food Chem. Toxicol.* 116(Pt B), 388–413. 10.1016/j.fct.2018.04.015 29654848

[B28] Phillips B. Szostak J. Titz B. Schlage W. K. Guedj E. Leroy P. (2019). A six-month systems toxicology inhalation/cessation study in ApoE(-/-) mice to investigate cardiovascular and respiratory exposure effects of modified risk tobacco products, CHTP 1.2 and THS 2.2, compared with conventional cigarettes. *Food Chem. Toxicol.* 126 113–141. 10.1016/j.fct.2019.02.008 30763686

[B29] Phillips B. Veljkovic E. Boue S. Schlage W. K. Vuillaume G. Martin F. (2016). An 8-Month systems toxicology Inhalation/Cessation Study in Apoe-/- Mice to investigate cardiovascular and respiratory exposure effects of a candidate modified risk tobacco Product, THS 2.2, compared with conventional cigarettes. *Toxicol. Sci.* 149 411–432. 10.1093/toxsci/kfv243 26609137 PMC4725610

[B30] Phillips B. Veljkovic E. Peck M. J. Buettner A. Elamin A. Guedj E. (2015). A 7-month cigarette smoke inhalation study in C57BL/6 mice demonstrates reduced lung inflammation and emphysema following smoking cessation or aerosol exposure from a prototypic modified risk tobacco product. *Food Chem. Toxicol.* 80 328–345. 10.1016/j.fct.2015.03.009 25843363

[B31] Png C. W. Linden S. K. Gilshenan K. S. Zoetendal E. G. McSweeney C. S. Sly L. I. (2010). Mucolytic bacteria with increased prevalence in IBD mucosa augment in vitro utilization of mucin by other bacteria. *Am. J. Gastroenterol.* 105 2420–2428. 10.1038/ajg.2010.281 20648002

[B32] Poussin C. Sierro N. Boué S. Battey J. Scotti E. Belcastro V. (2018). Interrogating the microbiome: experimental and computational considerations in support of study reproducibility. *Drug Discov. Today* 23 1644–1657. 10.1016/j.drudis.2018.06.005 29890228

[B33] Qin J. Li Y. Cai Z. Li S. Zhu J. Zhang F. (2012). A metagenome-wide association study of gut microbiota in type 2 diabetes. *Nature* 490 55–60. 23023125 10.1038/nature11450

[B34] R Core Team (2018). *R: A Language and Environment for Statistical Computing; 2015.* Geneva: R Core Team.

[B35] Santacruz A. Collado M. C. Garcia-Valdes L. Segura M. T. Martin-Lagos J. A. Anjos T. (2010). Gut microbiota composition is associated with body weight, weight gain and biochemical parameters in pregnant women. *Br. J. Nutr.* 104 83–92. 10.1017/s0007114510000176 20205964

[B36] Savin Z. Kivity S. Yonath H. Yehuda S. (2018). Smoking and the intestinal microbiome. *Arch. Microbiol.* 200 677–684. 10.1007/s00203-018-1506-2 29626219

[B37] Scotti E. Boué S. Sasso G. L. Zanetti F. Belcastro V. Poussin C. (2017). Exploring the microbiome in health and disease: implications for toxicology. *Toxicol. Res. Appl.* 1:2397847317741884.

[B38] Sirén J. Välimäki N. Mäkinen V. (2014). HISAT2-fast and sensitive alignment against general human population. *IEEE ACM Trans. Comput. Biol. Bioinforma* 11 375–388. 10.1109/TCBB.2013.2297101 26355784

[B39] Smith M. R. Clark B. Ludicke F. Schaller J. P. Vanscheeuwijck P. Hoeng J. (2016). Evaluation of the tobacco heating System 2.2. Part 1: description of the system and the scientific assessment program. *Regul. Toxicol. Pharmacol.* 81(Suppl. 2), S17–S26. 27450400 10.1016/j.yrtph.2016.07.006

[B40] Sommer F. Bäckhed F. (2013). The gut microbiota—masters of host development and physiology. *Nat. Rev. Microbiol.* 11 227–238. 10.1038/nrmicro2974 23435359

[B41] Tam A. Filho F. S. L. Ra S. W. Yang J. Leung J. M. Churg A. (2020). Effects of sex and chronic cigarette smoke exposure on the mouse cecal microbiome. *PLoS One* 15:e0230932. 10.1371/journal.pone.0230932 32251484 PMC7135149

[B42] Tilg H. Moschen A. R. (2014). Microbiota and diabetes: an evolving relationship. *Gut* 63 1513–1521. 10.1136/gutjnl-2014-306928 24833634

[B43] Turnbaugh P. J. Hamady M. Yatsunenko T. Cantarel B. L. Duncan A. Ley R. E. (2009). A core gut microbiome in obese and lean twins. *Nature* 457 480–484. 10.1038/nature07540 19043404 PMC2677729

[B44] Verschuere S. De Smet R. Allais L. Cuvelier C. A. (2012). The effect of smoking on intestinal inflammation: what can be learned from animal models? *J. Crohn’s Colitis* 6 1–12. 10.1016/j.crohns.2011.09.006 22261522

