# Supplementary material for: Impact of 6-Month Exposure to Aerosols From Potential Modified Risk Tobacco Products Relative to Cigarette Smoke on the Rodent Gastrointestinal Tract
Source: Front Microbiol. 2021 Jul 2;12:587745. doi: 10.3389/fmicb.2021.587745 (PMC8283309; doi:10.3389/fmicb.2021.587745)
Supplement: Supplementary file 1 [file Data_Sheet_1.docx]

# Supplementary figures


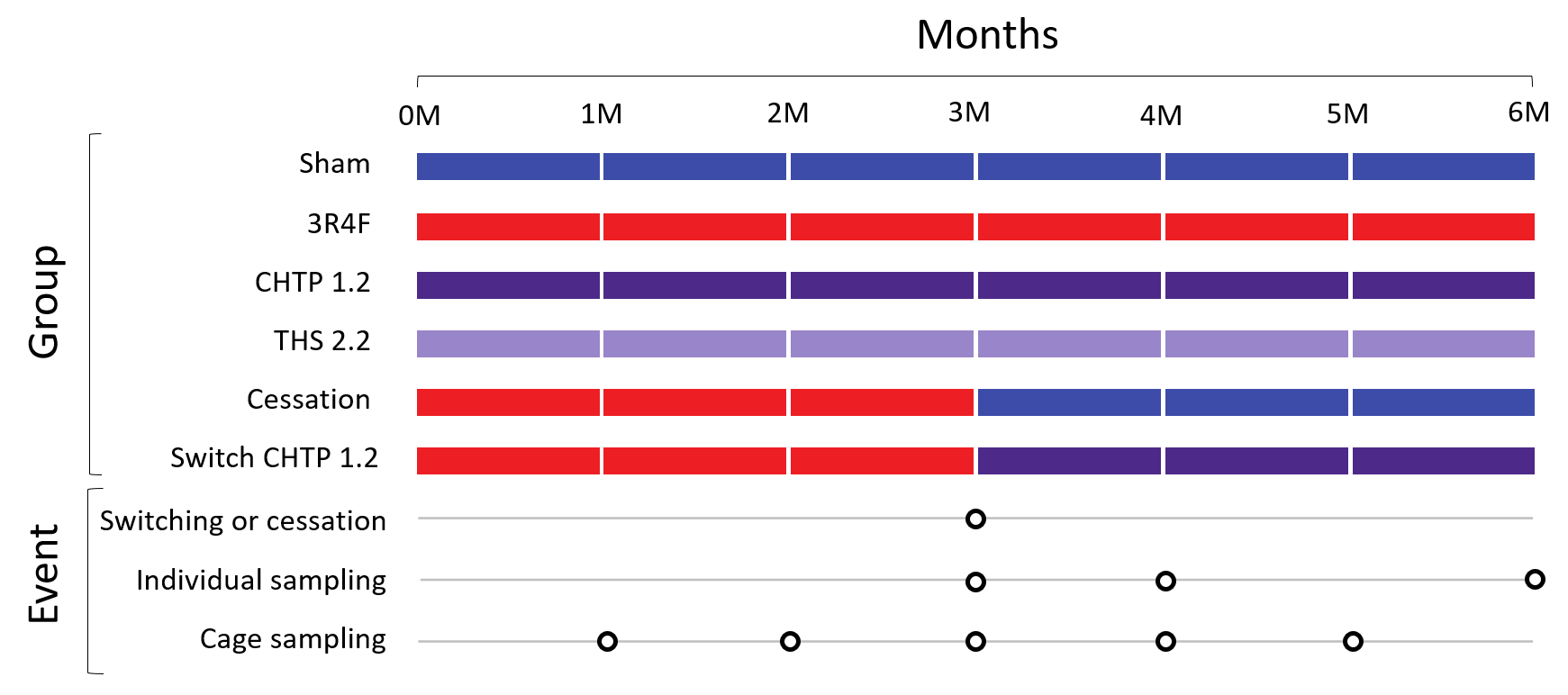


**Supplementary figure 1.** Experimental setup for aerosol exposure, illustrating the timepoints for individual and cage-wise sample collection.


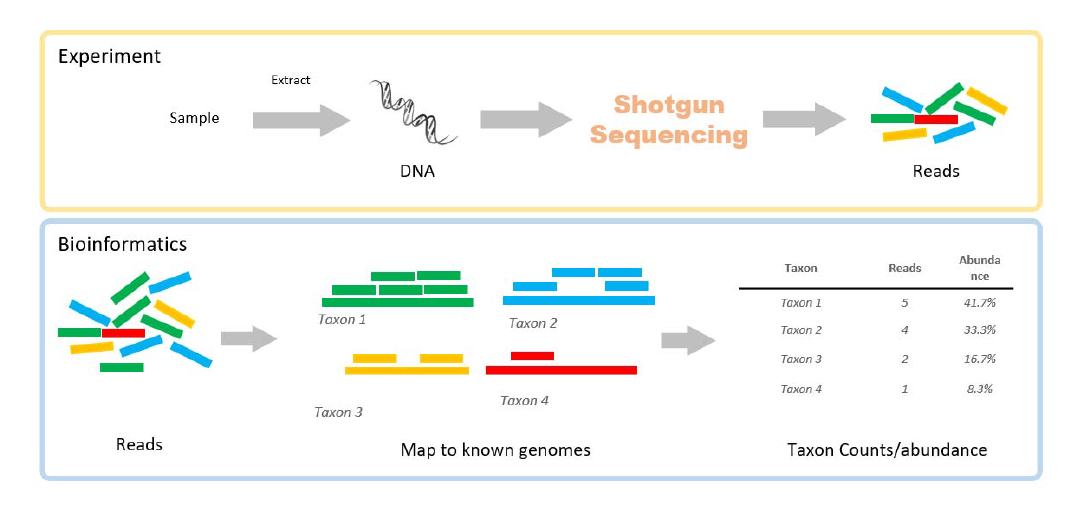


**Supplementary figure 2.** Schematic representation of the steps involved in the generation and processing of shotgun microbiome sequencing data.


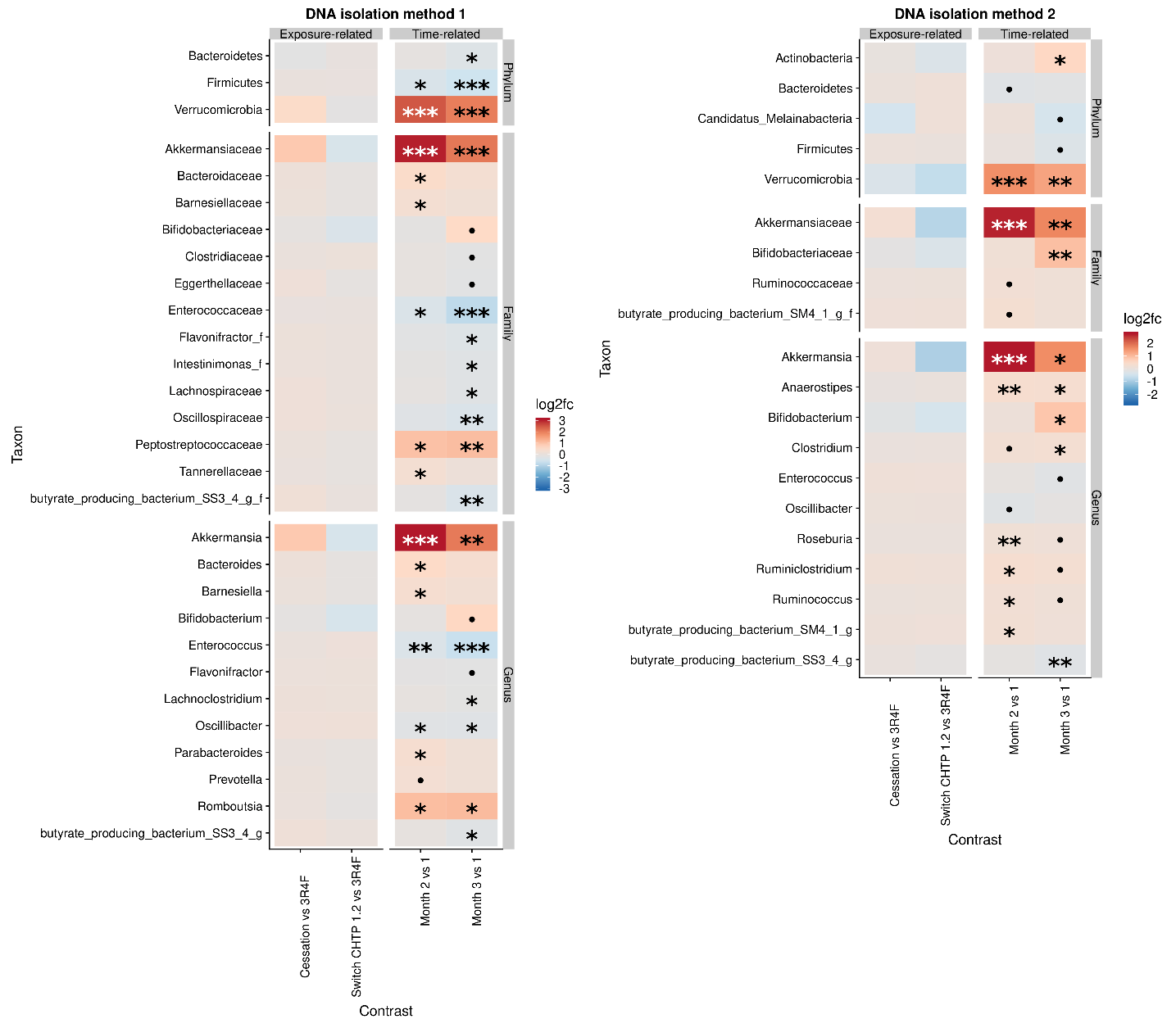


**Supplementary figure 3.** Short-term microbiome effects in groups initially exposed to 3R4F smoke. Significance values are indicated as follows: “***”: p <= 0.001; “**”: 0.001 < p <= 0.01, “*”: 0.01 < p <= 0.05; “.”: 0.05 < p <= 0.1.

Fecal samples

Cecal samples


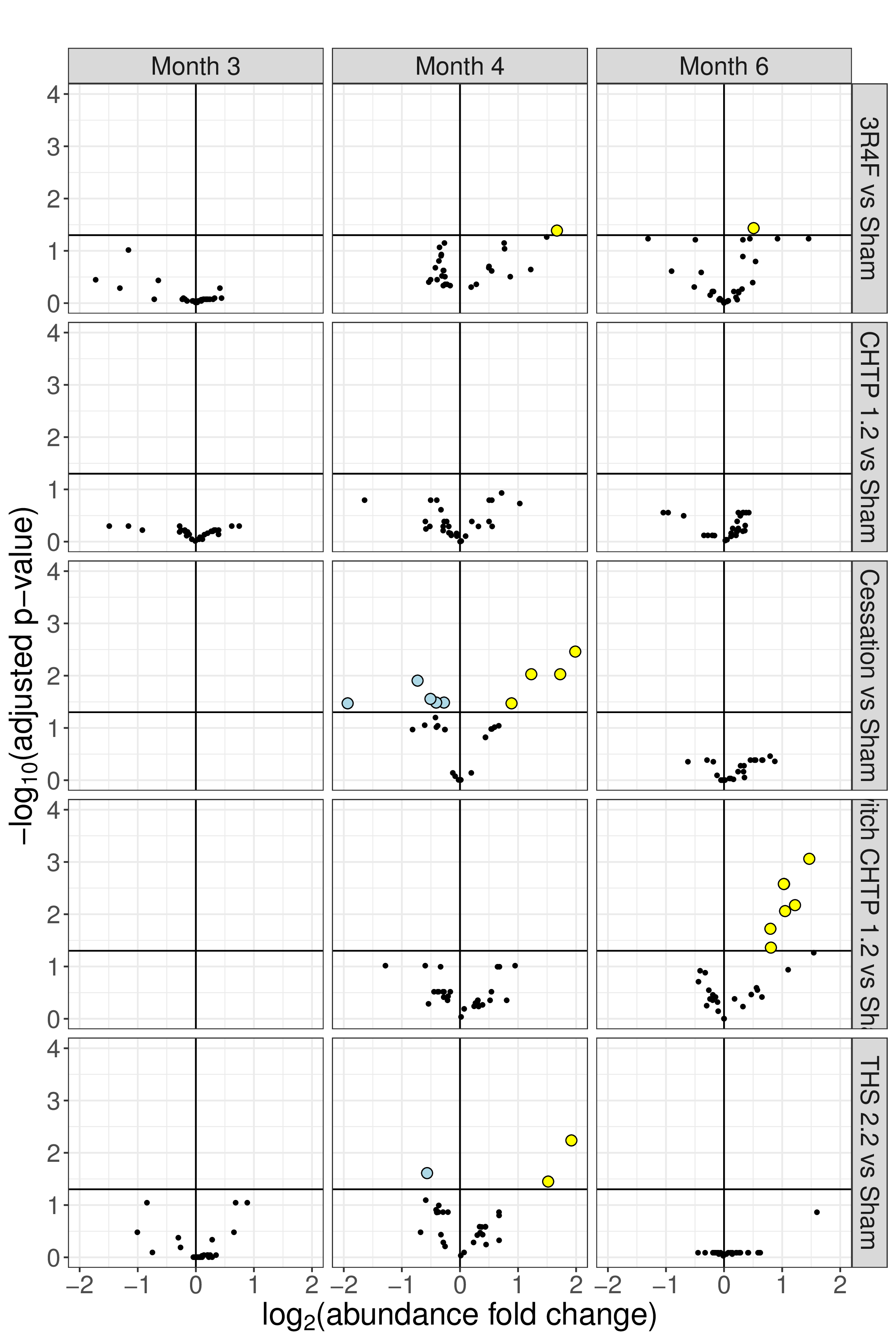

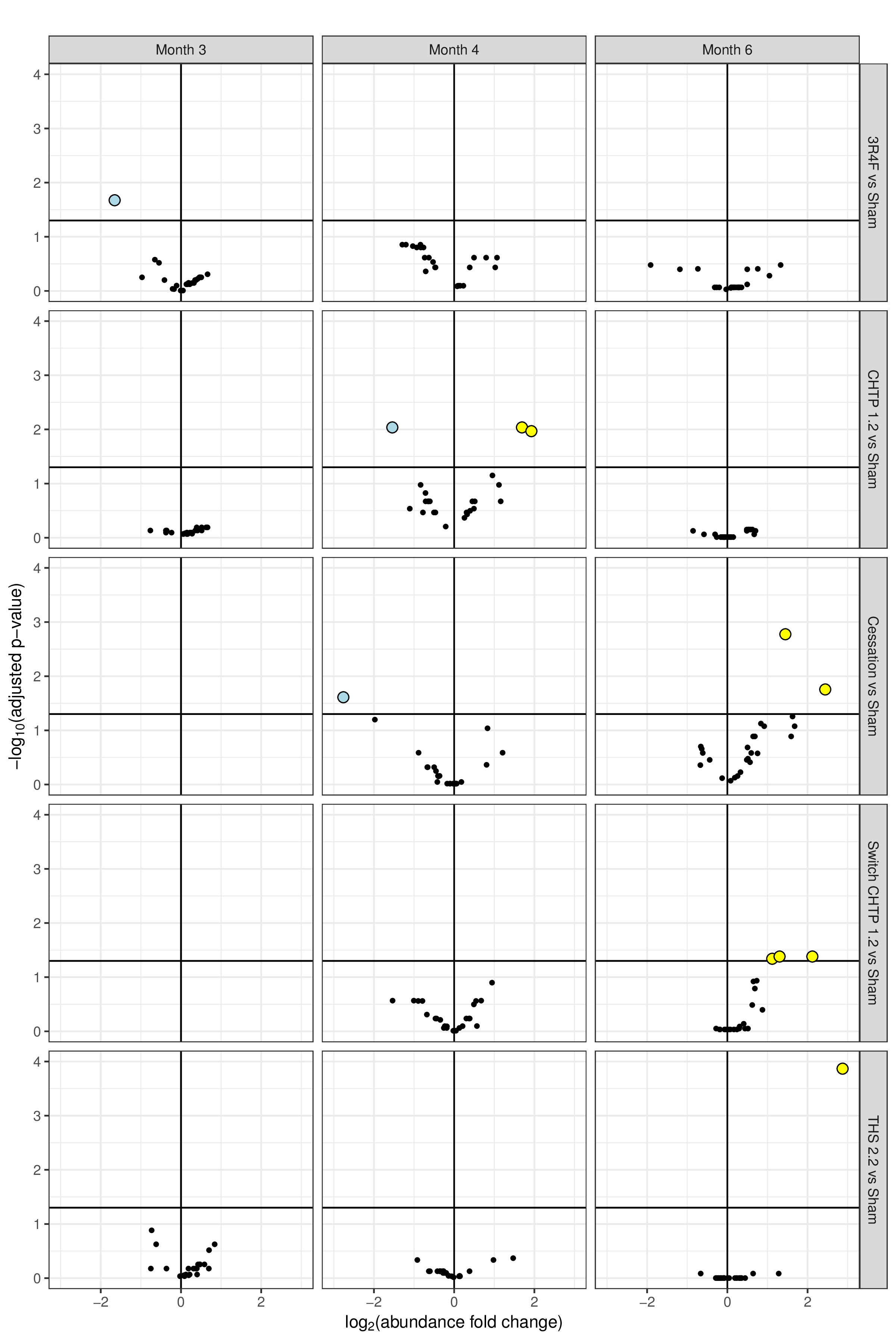


**Supplementary figure 4.** Differential abundance of bacterial families isolated from the cecal digesta and fecal matter of mice in the various exposure groups, displayed as a “volcano plot”. Each datapoint corresponds to a gene, with the x-axis value being the log2 fold change in the exposure group relative to the sham group, and the y-axis value being the negative log10 of the adjusted p value associated with the fold change. Points with adjusted p values <= 0.05 are rendered as large circles and colored by whether they have an expression fold change less than -2 (light blue) or greater than 2 (yellow).

# Supplementary tables

**Supplementary table 1**

| **Condition** |  | **Time point (Months)** | | | | |
| --- | --- | --- | --- | --- | --- | --- |
|  |  | **1M** | **2M** | **3M** | **4M** | **5M** |
| 3R4F |  | 11 | 11 | 11 | 9 | 9 |
| CHTP |  | 8 | 8 | 8 | 6 | 6 |
| Cess |  | 11 | 11 | 11 | 9 | 9 |
| Sham |  | 8 | 8 | 8 | 6 | 6 |
| SwitchCHTP |  | 10 | 10 | 10 | 10 | 10 |
| THS |  | 8 | 8 | 8 | 6 | 6 |

**Supplementary table 2.**

| **Condition** |  | **Sample Type** |  | **Time point (Months)** | | |
| --- | --- | --- | --- | --- | --- | --- |
|  |  |  |  | **3M** | **4M** | **6M** |
| 3R4F |  | cecum |  | 9 | 9 | 9 |
| 3R4F |  | digesta,cecal |  | 9 | 9 | 9 |
| 3R4F |  | excreta |  | 9 | 9 | 9 |
| CHTP |  | cecum |  | 9 | 9 | 9 |
| CHTP |  | digesta,cecal |  | 9 | 9 | 9 |
| CHTP |  | excreta |  | 9 | 9 | 9 |
| Cessation |  | cecum |  | N/A | 9 | 9 |
| Cessation |  | digesta,cecal |  | N/A | 9 | 9 |
| Cessation |  | excreta |  | N/A | 9 | 9 |
| Sham |  | cecum |  | 9 | 9 | 9 |
| Sham |  | digesta,cecal |  | 9 | 9 | 9 |
| Sham |  | excreta |  | 9 | 9 | 9 |
| Switch-CHTP |  | cecum |  | N/A | 9 | 9 |
| Switch-CHTP |  | digesta,cecal |  | N/A | 9 | 9 |
| Switch-CHTP |  | excreta |  | N/A | 9 | 9 |
| THS |  | cecum |  | 9 | 9 | 9 |
| THS |  | digesta,cecal |  | 9 | 9 | 9 |
| THS |  | excreta |  | 9 | 9 | 9 |

**Supplementary table 3**

| Correlation of normalized read counts from fecal and cecal samples of the sample animals | |
| --- | --- |
| Taxon | R |
| *Akkermansiaceae* | 0.85 |
| *Erysipelotrichaceae* | 0.80 |
| *Candidatus_Melainabacteria_bacterium_MELA1_g_f* | 0.78 |
| *Bifidobacteriaceae* | 0.72 |
| *Peptostreptococcaceae* | 0.63 |
| *Bacteroidaceae* | 0.56 |
| *Flavobacteriaceae* | 0.55 |
| *butyrate_producing_bacterium_SM4_1_g_f* | 0.52 |
| *Enterococcaceae* | 0.47 |
| *butyrate_producing_bacterium_SS3_4_g_f* | 0.44 |
| *Prevotellaceae* | 0.40 |
| *Eubacteriaceae* | 0.36 |
| *Barnesiellaceae* | 0.36 |
| *Tannerellaceae* | 0.32 |
| *Clostridiaceae* | 0.30 |
| *Rikenellaceae* | 0.29 |
| *Oscillospiraceae* | 0.28 |
| *Ruminococcaceae* | 0.27 |
| *Muribaculaceae* | 0.26 |
| *Flavonifractor_f* | 0.26 |
| *Eggerthellaceae* | 0.26 |
| *Lactobacillaceae* | 0.22 |
| *Lachnospiraceae* | 0.22 |
| *Intestinimonas_f* | 0.21 |
